# *Klebsiella pneumoniae *triggers a cytotoxic effect on airway epithelial cells

**DOI:** 10.1186/1471-2180-9-156

**Published:** 2009-08-03

**Authors:** Victoria Cano, David Moranta, Enrique Llobet-Brossa, José Antonio Bengoechea, Junkal Garmendia

**Affiliations:** 1 Fundación Caubet-CIMERA, Programa de Infección e Inmunidad, Recinto Hospital Joan March, carretera Sóller, km 12, 07110, Bunyola, Spain; 2 Centro de Investigación Biomédica en Red de Enfermedades Respiratorias (CIBERES), Bunyola, Spain; 3 Área Microbiología, Facultad de Biología, Universidad Illes Balears, Carretera Valldemossa, km 7.5, 07122, Palma de Mallorca, Spain; 4 Consejo Superior de Investigaciones Científicas (CSIC), Madrid, Spain

## Abstract

**Background:**

*Klebsiella pneumoniae *is a capsulated Gram negative bacterial pathogen and a frequent cause of nosocomial infections. Despite its clinical relevance, little is known about the features of the interaction between *K. pneumoniae *and lung epithelial cells on a cellular level, neither about the role of capsule polysaccharide, one of its best characterised virulence factors, in this interaction.

**Results:**

The interaction between *Klebsiella pneumoniae *and cultured airway epithelial cells was analysed. *K. pneumoniae *infection triggered cytotoxicity, evident by cell rounding and detachment from the substrate. This effect required the presence of live bacteria and of capsule polysaccharide, since it was observed with isolates expressing different amounts of capsule and/or different serotypes but not with non-capsulated bacteria. Cytotoxicity was analysed by lactate dehydrogenase and formazan measurements, ethidium bromide uptake and analysis of DNA integrity, obtaining consistent and complementary results. Moreover, cytotoxicity of non-capsulated strains was restored by addition of purified capsule during infection. While a non-capsulated strain was avirulent in a mouse infection model, capsulated *K. pneumoniae *isolates displayed different degrees of virulence.

**Conclusion:**

Our observations allocate a novel role to *K. pneumoniae *capsule in promotion of cytotoxicity. Although this effect is likely to be associated with virulence, strains expressing different capsule levels were not equally virulent. This fact suggests the existence of other bacterial requirements for virulence, together with capsule polysaccharide.

## Background

*Klebsiella pneumoniae *is the most common Gram-negative bacterium causing community-acquired pneumonia and up to 5% of community-acquired urinary tract infections [1-3]. Community-acquired pneumonia is a very severe illness with a rapid onset, and despite the availability of an adequate antibiotic regimen, the outcome is often fatal. The observed mortality rates are about 50% [4]. Capsule polysaccharide (CPS), siderophores, lipopolysaccharide (LPS) and adhesins are virulence factors identified for this pathogen. However, most of the studies have focused on the role of CPS in *Klebsiella *virulence. Early studies suggested that an extracellular toxic complex mainly composed of CPS triggers extensive lung tissue damage [5,6] and data indicate that there might be a correlation between the production of this extracellular complex and *Klebsiella *virulence [5,6]. Similar to CPSs from other pathogens, *Klebsiella *CPS is responsible for resistance to complement mediated killing [7] and impedes adhesion to and invasion of epithelial cells [8] by sterically preventing receptor-target recognition of bacterial adhesins [9,10]. Recently we have demonstrated that CPS mediates resistance to antimicrobial peptides (APs), trapping APs and thus acting as a bacterial decoy [11,12].

Few studies have analysed cellular features of the interaction between lung epithelium and *K. pneumoniae*, the role of virulence factors such as CPS, and the relevance of this interaction *in vivo*. We have recently shown that an isogenic CPS mutant activates host cellular inflammatory responses and that CPS might prevent this activation through blockage of bacterial uptake [13]. Moreover, *Klebsiella *infection increases the expression levels of Toll-like receptors 2 and 4 (TLR2 and TLR4) [14]. This increased expression of TLRs results in an enhancement of the cellular response upon stimulation with Pam3CSK4 or lipopolysaccharide, TLR2 and TLR4 agonists, respectively [14]. In this study, we show for the first time that *K. pneumoniae *exerts a cytotoxic effect on airway epithelial cells that is associated with the presence of CPS.

## Methods

### Bacterial strains

*K. pneumoniae *strains 52145 and 1850 are clinical isolates belonging to serotypes O1:K2 and O1:K35, respectively [15]. *K. pneumoniae *strain 43816 (ATCC 43816) belongs to serotype O1:K2. *K. pneumoniae *52K10 is a derivative of strain 52145 which lacks CPS [16]. *K. pneumoniae *strains were cultured in Luria-Bertani (LB) medium at 37°C.

### CPS purification and quantification

Cell-bound CPS was purified by the phenol-water method [17]. Briefly, bacteria were grown in 1 l LB-broth in 2 l flasks in an orbital shaker (180 rpm) for 24 h at 37°C. Cells were removed by centrifugation and washed once with PBS. The pellet was extracted with phenol, and polysaccharides present in the aqueous phase were precipitated by adding 5 volumes of methanol plus 1% (v/v) of a saturated solution of sodium acetate in methanol. After incubation for 24 h at -20°C, the pellet was recovered by centrifugation, dissolved in distilled water, dialysed against water and freeze-dried. For further purification, this preparation was dispersed (final concentration 10 mg/ml) in 0.8% NaCl/0.05% NaN_3_/0.1 M Tris-HCl (pH 7) and digested with nucleases (50 mg/ml of DNase II type V and RNase A [Sigma Chemical Co., St. Louis, Mo.]) for 18 h at 37°C. Proteinase K was added (50 mg/ml [E. Merck, Darmstadt, Germany]), and the mixture was incubated for 1 h at 55°C and for 24 h at room temperature. The proteinase K digestion was repeated twice and the polysaccharides were precipitated as described above. The pellet was recovered by centrifugation and dissolved in distilled water. LPS was removed by ultracentrifugation (105000 × g, 16 h, 4°C) and samples were freeze-dried. The enzymatic treatment and ultracentrifugation steps were repeated once. This CPS preparation was repurified by the method described by Hirschfeld and co-workers [18]. This method is widely used to remove proteins from polysaccharide preparations. SDS-PAGE-resolved preparations were transferred to PVDF membrane which was stained with colloidal gold to visualize proteins [19]. No trace of contaminant proteins was found (data not shown). CPS was quantified by determining the concentration of uronic acid in the samples, using a modified carbazole assay [20] as described by Rahn and Whitfield [21]. LPS presence was determined by measuring the 3-deoxy-d-manno-2-octulosonic acid (Kdo) content by the thiobarbituric acid method modified to correct interference due to deoxysugars [22]. Kdo content was less than 0.07%.

### Mammalian cell culture and bacterial infection

Monolayers of human lung carcinoma cells (A549, ATCC CCL185) derived from type II pneumocytes were grown to confluence as described before [13]. Cells were serum starved for 18 h before infection. Overnight-grown bacteria were subcultured and grown to exponential phase, harvested by centrifugation (20 min/2700 × g) and resuspended in PBS. The inoculum for the infection was prepared in Earle's buffered salt solution (EBSS), pH 7.4. A549 cells (80–90% confluent) seeded on glass coverslips in 24-well tissue culture plates were subsequently infected with *K. pneumoniae *strains at a multiplicity of infection (MOI) ranging from 100:1 to 1000:1 and centrifuged for 4 min at 200 × g at 22°C. Infected plates were then incubated for 2 to 5 h at 37°C/5% CO_2 _in a humidified incubator. For adhesion assays, cells were washed five times with 1 ml phosphate-buffered saline (PBS) pH 7.4 after 2 h of infection and lysed with 0.5%-Triton in PBS. Serial dilutions of the lysates in PBS were plated on LB plates for quantification of viable bacteria. Experiments were carried out in triplicate in three independent occasions and results are expressed as % adhesion = 100 × (n° of bacteria recovered from well/initial n° of bacteria added). Where indicated, bacteria were UV killed by exposure to 1 joule for 3 min in a BIO-LINK BLX crosslinker (Vilber Lourmat).

### Fluorescence microscopy

Cell monolayers were fixed in 3.7% paraformaldehyde in PBS. Rhodamine (RRX)-conjugated phalloidin (Molecular Probes) diluted 1:200 in 10% horse serum/0.1% saponin in PBS was used to stain the actin cytoskeleton. Coverslips were washed twice in PBS containing 0.1% saponin, once in PBS, and incubated for 30 min with phalloidin-RRX. The coverslips were then washed twice in 0.1% saponin in PBS, once in PBS and once in H_2_O, mounted in Aqua-Poly/Mount (Polysciences) and analysed with a Leica CTR6000 fluorescence microscope.

### Analysis of host cell DNA integrity after *K. pneumoniae *infection

A549 cells were infected with *K. pneumoniae *strains at MOI of 500:1 in tissue culture plates. 6 h post-infection, cells (~2.5 × 10^6^) from 2 wells were collected in PBS by scraping and lysed in 600 μl cold lysis buffer (10 mM Tris-HCl pH 8, 1 mM EDTA, 0.1% SDS). Proteinase K (100 μg/ml) was added and samples were incubated for 3 h at 55°C. Samples were cooled to 22°C and incubated with 20 μg/ml RNase (DNase-free) for 20 min at 37°C. 200 μl 5 M potassium acetate were added and samples were centrifuged (13000 × rpm, 22°C, 1 min). DNA present in the supernatants was precipitated with isopropanol, washed in 70% ethanol and dissolved in sterile water. DNA integrity was analysed by staining with ethidium bromide after resolving the samples by gel-electrophoresis in 1% agarose in TAE.

### Cell cytotoxicity and viability assays

A549 cells (cultured in either 24- or 96-well plates) were infected with *K. pneumoniae *strains (MOI 500:1 or 1000:1, 5 h). Lactate dehydrogenase (LDH) release was measured using a commercial kit (CytoTox 96, Promega). Per cent cytotoxicity was calculated as: (OD_490 _sample - OD_490 _medium)/(OD_490 _max - OD_490 _medium)*100. OD_490 _max was obtained with the provided lysis positive control. Measure of formazan production from reduction of MTS tetrazolium by metabolically active cells was performed using cells cultured in 96-well plates. Formazan production (% viability) was measured using a kit (CellTiter 96 AQueous One, Promega) and calculated as: OD_490 _sample/OD_490 _max*100. OD_490 _max was obtained from a monolayer of non-infected cells. Ethidium bromide is taken up by host cells when cytoplasmic membrane integrity is lost, staining nuclei red when visualised by fluorescence microscopy. Cells were cultured on coverslips in 24-well plates and infected as described above (MOI 500:1, 5 h). 15 min before the end of the infection, culture medium was removed and wells were washed with 1 ml PBS. Cells were stained for 10 min with 250 μl of 6 TM ethidium bromide prepared in PBS, washed three times with 1 ml PBS, fixed with 3.7% paraformaldehyde in PBS, and mounted for immunofluorescence analysis as described above. Cytotoxicity (red nuclei) was quantified by counting a minimum of 100 cells in three independent experiments.

### Mouse pneumonia model

Overnight-grown bacteria were subcultured and grown to exponential phase. Bacteria were centrifuged (2500 × g, 20 min, 22°C), resuspended in PBS and adjusted to 5 × 10^6 ^colony-forming units (c.f.u.)/ml. Five to seven-week-old female C57BI/6j mice were anaesthetized by i.p. injection with a mixture containing ketamine (100 mg/ml) and xylazine (10 mg/ml). 20 μl of bacterial suspension were inoculated intranasally in 4 × 5 μl aliquots. 48 or 72 h post-infection the mice were sacrificed by cervical dislocation and trachea, spleen and liver were dissected, weighed and homogenized in 1 ml PBS. Serial dilutions of the homogenates in PBS were plated on LB agar to determine c.f.u. per gram of tissue.

### Statistics

Statistical analyses were performed with Prism4 for PC (GraphPad Software) using the analysis of variance (ANOVA) or the two-sample *t *test or, when the requirements were not met, by the Mann-Whitney U test. *P *< 0.05 was considered statistically significant.

## Results

### *K. pneumoniae *induces a cytotoxic effect in lung epithelial cells

**Figure 1:**
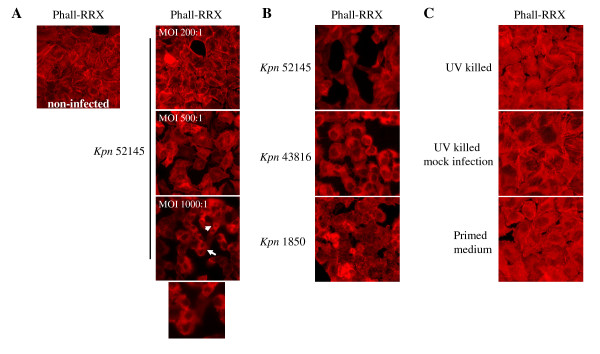
***K. pneumoniae *triggers a cytotoxic effect during infection of A549 carcinoma lung epithelial cells**. **A**. Infection of A549 lung epithelial cells with *K. pneumoniae *52145. MOIs used were 200:1 (top), 500:1 (middle) and 1000:1 (bottom panel and detail). Infections were carried out for 5 h in all cases. Non infected cells are shown for comparison (top left). Cells were fixed and stained for immunofluorescence. Actin cytoskeleton was labelled with phalloidin-RRX (red). White arrows show cell rounding and cytotoxicity. **B**. A549 epithelial cells were infected with *K. pneumoniae *strains 52145, 43816 and 1850 at MOI 500:1 for 4 h. Infected cells were fixed and stained with phalloidin-RRX for immunofluorescence as indicated above. **C**. UV killed *K. pneumoniae *52145 was used to infect cells at MOI 500:1 during 4 h (top). *K. pneumoniae *52145 was used for a mock infection (MOI 500:1). After 4 h the bacterial suspension was UV irradiated and used to infect a confluent cell monolayer for 4 h (middle). To assess the need of presence of live bacteria to induce cell rounding, infection was carried out at MOI 500:1 during 4 h, after which the supernatant was collected, centrifuged and filtered (0.2 Tm, nitrocellulose) to obtain a primed bacteria-free medium, which was then added to a new epithelium monolayer for 4 h (bottom). Infected cells were fixed and stained for immunofluorescence as described above.

A549 lung epithelial cells were infected with *K. pneumoniae *52145 (52145), a highly capsulated strain (339 μg per 10^5 ^c.f.u.) for 5 h with different MOIs and the host actin cytoskeleton was stained. This is a sensitive method to detect whether a pathogen induces cytoskeleton disturbances which can be associated with host cell cytotoxicity [23]. Actin cytoskeleton staining revealed that cells rounded up at MOI 500:1 (Fig. 1A), followed by detachment from the substrate. At MOI 1000:1 a cytotoxic effect on the cell monolayer was observed (white arrows and detail). To determine the minimal infection requirements for cell rounding, cells were infected with different MOIs for 2 to 5 h. Cell rounding was observed when cells were infected at MOI 500:1 for 4 h (Fig. 1B, top). To investigate whether the cytotoxic effect was strain dependent two additional *K. pneumoniae *strains were tested. Strains 43816 (serotype K2) and 1850 (serotype K35) also induced cell rounding (Fig. 1B, middle and bottom, respectively). CPS amounts expressed by these strains, 238 and 35 μg per 10^5 ^c.f.u., respectively, are lower than that expressed by strain 52145 (339 μg per 10^5 ^c.f.u.), indicating that *Klebsiella*-induced cytotoxicity is not absolutely dependent on the amount of CPS expressed.

Next, we asked whether live bacteria are necessary to induce cell rounding. The bacterial inoculum was killed by UV radiation and used to infect cells (MOI 500:1, 4 h). Under these conditions, strain 52145 did not induce cell rounding (Fig. 1C, upper). In order to corroborate this observation, a mock infection was carried out, i.e. same infection conditions as before, but in a tissue culture well without cells. After 4 h, the bacterial suspension was UV irradiated and used to infect a confluent cell monolayer for 4 h. Cell rounding was not observed (Fig. 1C, middle). In addition, the strain 52145-triggered cytotoxic effect was not induced by primed bacteria-free conditioned medium, since A549 monolayers remained intact after 4 h of exposure to bacteria-free medium obtained from previously infected cells (MOI 500:1, 4 h) (Fig. 1C, lower). Taken together, these findings demonstrate that *K. pneumoniae *strain 52145 induces a cytotoxic effect through a process requiring the presence of live bacteria.

### *K. pneumoniae*-induced cytotoxicity is dependent on the presence of CPS

**Figure 2:**
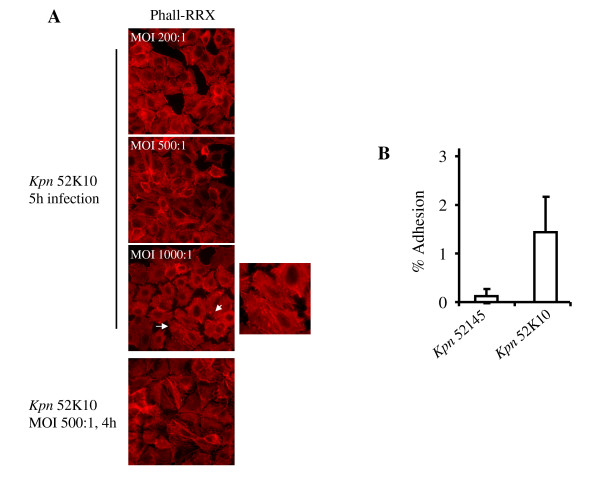
**Capsule polysaccharide (CPS) is required for cytotoxicity during *K. pneumoniae *infection of A549 lung epithelium**. **A**. Infection of A549 lung epithelial cells with *K. pneumoniae *52K10, a bacterial strain lacking CPS. MOIs used were 200:1 (upper), 500:1 (middle) and 1000:1 (lower panel and right detail). Infections were carried out for 5 h in all cases. Infection conditions of MOI 500:1 for 4 h were used in the bottom panel. Infected cells were fixed and stained for immunofluorescence. Actin cytoskeleton was labelled with phalloidin-RRX (red). White arrows and detail show cell spread morphology and absence of cytotoxicity. **B**. Adhesion levels of *K. pneumoniae *strains 52145 and 52K10 to A549 lung epithelial cells. Infections were carried out at MOI 100:1 for 2 h. Mean values from three independent experiments are shown (error bars = SD).

We sought to pinpoint bacterial factor(s) responsible for strain 52145-triggered cytotoxicity. Taken into account that several studies have demonstrated the important role of CPS in the interplay between *K. pneumoniae *and eukaryotic host cells, we asked whether CPS might play a role in the *Klebsiella*-induced cytotoxicity. We studied whether an isogenic CPS mutant of 52145, strain 52K10 [16], would induce cytotoxicity. Immunofluorescence analysis of the actin cytoskeleton of infected A549 cells showed that strain 52K10 did not induce cytotoxicity under all conditions tested, hence suggesting that CPS could be one of the bacterial factors involved in 52145-triggered cytotoxicity (Fig. 2A). Furthemore, the lack of cytotoxicity during 52K10 infection was not due to a decrease in bacterial adhesion levels because 52K10 adhesion levels to A549 cells were actually higher than those displayed by CPS-expressing strains (Fig. 2B). Even though cytotoxicity by non-capsulated strain was at some extent promoted by addition of purified CPS during infection, purified CPS alone did not trigger a cytotoxic effect (data not shown), suggesting that additional bacterial elements besides CPS may contribute to cytotocixity during *K. pneumoniae *infection.

**Figure 3:**
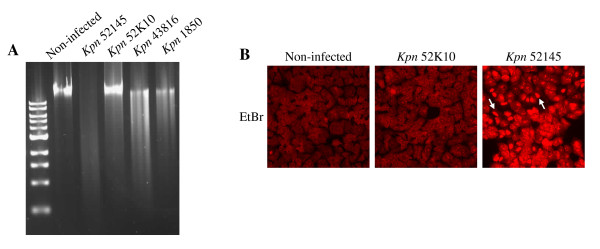
***Klebsiella *induced cytotoxicity is observed by disintegration of host genomic DNA and loss of host plasma membrane integrity**. **A**. Ethidium bromide staining after agarose gel-electrophoresis of genomic DNA isolated from A549 epithelial cells infected with *K. pneumoniae *strains 52145, 43816, 1850 or 52K10. **B**. A549 lung epithelial cells were not infected (left), infected with *K. pneumoniae *strain 52K10 (middle), or strain 52145 (right). The cells were stained with ethidium bromide and analysed by fluorescence microscopy. Necrotic or apoptotic cells had normal/condensed nuclei that were brightly stained with ethidium bromide and appeared red (white arrows).

To further characterize the cytotoxic effect induced by 52145, cell toxicity was assessed by four independent methods: (i) lactate dehydrogenase (LDH) release, (ii) production of formazan, (iii) analysis of DNA integrity, and (iv) uptake of ethidium bromide. LDH release, taken as an indicator of host cell membrane integrity and cell viability, was measured in supernatants of cells infected with strains 52145 or 52K10, and compared to that released by non-infected cells (see Methods section). *K. pneumoniae *strain 52145 (MOI 500:1, 5 h) triggered 30.2 ± 0.28% cytotoxicity, which was approximately 1.5 times higher than that induced by strain 52K10 (20.2 ± 2.19%). Formazan is produced by reduction of MTS tetrazolium by metabolically active cells and thus serves as an indicator of cell viability. Formazan production (% viability) was lower in strain 52145-infected cells (32.9 ± 6.5%) than in non-infected (100%) or 52K10-infected cells (134 ± 4.9%). DNA fragmentation is taken as a sign of cell death by apoptosis. A prominent DNA laddering/degradation could be seen after 6 h of infection with *K. pneumoniae *strains 52145, 43816 and 1850 (Fig. 3A). However, DNA extracted from cells infected with strain 52K10 was intact, similar to DNA obtained from non-infected cells (Fig. 3A). Finally, we analysed the uptake of ethidium bromide by infected cells. Ethidium bromide is taken up by the cells only when integrity of the plasma membrane is lost. Red fluorescence staining of nuclei is therefore an indicator of plasma membrane integrity loss. The percentage of cells which had taken up the dye was higher in 52145-infected cells (21.2 ± 2.2%) than in 52K10-infected cells (1.74 ± 0.9%) or in non-infected cells (0%). Representative pictures are shown in Fig. 3B.

In summary, these findings indicate that *K. pneumoniae *alters host cell viability in a process dependent on the presence of CPS.

### Correlation between *K. pneumoniae*-induced cell cytotoxicity and virulence

**Figure 4:**
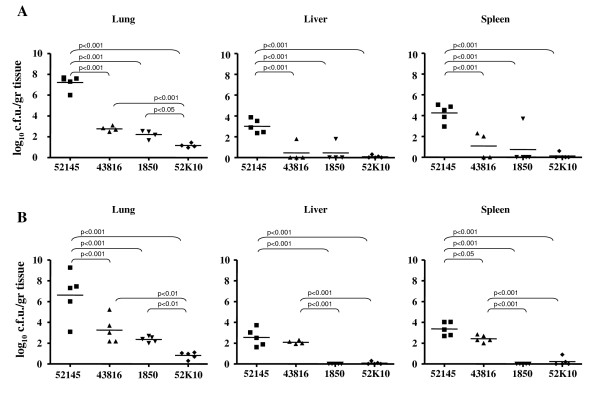
**Mouse pneumonia model for *K. pneumoniae *strains**. Intranasal infections by *K. pneumoniae *strains 52145, 43816, 1850 and 52K10. Mice were infected with 10^5 ^c.f.u. and sacrificed 48 h (**A**) or 72 h (**B**) post-infection. Lung, spleen and liver were dissected, weighed, homogenized and plated on LB agar. Data shown are from five infected mice per time point. Mean values are plotted.

It is well known that CPS is essential for *K. pneumoniae*-induced pneumonia [16] and we have established here that *Klebsiella*-induced cytotoxicity depends on the presence of CPS. We sought then to determine whether induction of cytotoxicity is sufficient for *K. pneumoniae *virulence using an intranasal model of infection. As an infection marker, we determined the bacterial loads in lung, liver and spleen for *K. pneumoniae *strains 52145, 43816, 1850. Strain 52145 successfully infected mouse lungs (Fig. 4A and 4B, left) and disseminated to liver (Fig. 4A and 4B, middle) and spleen (Fig. 4A and 4B, right). No decrease in the bacterial load, which was higher in lung than in liver and spleen, was observed in any organ at 72 h post-infection. Strains 43816 and 1850, both inducing cytotoxicity, displayed an intermediate virulence phenotype. Strain 43816 was detected in lungs, with similar recovery at 48 and 72 h post-infection. Systemic infection was delayed until 72 h post-infection. Strain 1850 was equally recovered from lungs at 48 and 72 h post-infection. Spleen and liver colonization were hardly observed at any time. As a control, we determined the bacterial loads in lung, liver and spleen of the CPS mutant strain 52K10. As reported previously [16], this mutant was attenuated. Viable counts recovered from lung were significantly lower than those for capsulated strains at 48 and 72 h post-infection and bacteria could not be recovered from liver or spleen at any time post-infection.

Therefore, although cytotoxicity is likely to be associated with virulence, strains expressing different capsule levels were not equally virulent, suggesting that additional bacterial factors could be involved in virulence, or that the cytotoxic effect is necessary, but not sufficient, for virulence.

## Discussion

In this study, we show that *K. pneumoniae *triggers a cytotoxic effect upon infection of human lung epithelial cells. This process requires the presence of capsulated live bacteria through the time of infection. To the best of our knowledge, there are no studies reporting that *K. pneumoniae *might exert a cytotoxic effect on airway epithelial cells. Our results could point to the underlying mechanism behind the early findings reported by Straus et al., [5,24] which indicated that *K. pneumoniae *expressing CPS induces extensive lung tissue damage.

A number of bacterial pathogens induce cytotoxicity in eukaryotic cells, which is frequently dependent on an active type IIIsecretion system (T3SS). For example, enteropathogenic *Escherichia coli *induces detachment of infected epithelial cells from the substratum and injects the T3SS effector Cif into cells, which induces a cytopathic effect [25,26]. *Bordetella bronchiseptica*'s necrotic effect on epithelial cells is dependent on the T3SS effector BopB [27], and also *Pseudomonas aeruginosa *promotes T3SS-dependent cytotoxicity towards eukaryotic cells [28,29]. Yet, *K. pneumoniae*-induced cytotoxicity does not seem to be related to a T3SS, given that *in silico *analysis of the so far sequenced *K. pneumoniae *genomes does not identify any T3SS components. Furthermore, PCR analysis using degenerated primers to amplify *lcrD *homologues present in all known T3SS were negative in all our *Klebsiella *strains. Recently, it has been shown that *P. aeruginosa *and enterotoxigenic *E. coli *deliver toxins directly into host cell cytoplasm using outer membrane vesicles [30,31]. It is likely that *K. pneumoniae *also produces outer membrane vesicles. In fact, the extracellular toxic complex described by Straus [5,24] could be considered a preparation of outer membrane vesicles. It is then tempting to speculate that outer membrane vesicles could be associated with *K. pneumoniae *cytotoxicity described in our study. Future studies will aim to address this possibility. On the other hand, our results clearly establish that CPS is necessary for the induction of cytotoxicity. CPS is a virulence factor for several pathogens, including *Streptococcus pneumoniae*, *Neisseria meningitidis*, *Haemophilus influenzae *type b and *E. coli *K1 [32-34]. Of note, no previous reports link the presence of CPS to cytotoxicity. However, just the presence of CPS is not sufficient for *K. pneumoniae*-induced cytotoxicity because capsulated UV-killed bacteria or purified CPS did not induce this effect. Given the limited current knowledge about *K. pneumoniae *virulence factors, we can only speculate on the nature of bacterial factor(s) that, together with CPS, could promote cytotoxicity in the host. Signature-tagged mutagenesis approaches have identified several virulence factors [35,36], but none of them resemble those triggering the cytotoxicity by other bacterial pathogens.

All *K. pneumoniae *clinical isolates are capsulated, inferring the importance of CPS for virulence. Likewise, CPS is necessary for virulence in an *in vivo *pneumonia model [15,35] and for *Klebsiella*-induced cytotoxicity (this work). However, our data indicate that CPS-dependent cytoxicity is necessary but not sufficient for *Klebsiella *virulence because strains 43816 and 1850 are less virulent than strain 52145 and the three of them trigger cytotoxicity. This could be explained by differences in the amount of CPS expressed by these strains, although strain 43816 is also considered to be heavily capsulated. The absence of complete correlation between *in vitro *and *in vivo *studies has been previously described for other *K. pneumoniae *isolates. Struve et al., showed that CPS expression reduced *K. pneumoniae *adhesion to gut and bladder epithelium, when compared to a noncapsulated mutant. However, the presence/absence of CPS had no effect on the colonisation of the gastrointestinal tract, but did play a role in colonisation of the urinary tract [37]. On the other hand, it has been recently postulated that there is an association between CPS serotype, virulence in mice and humans, and frequency of isolation in clinical settings [38]. However, the bacterial strains tested in this study express CPS belonging to serotypes considered to have high potential of causing disease [38], and strains 52145 and 43816 express the same CPS serotype. Nevertheless, *Klebsiella *infections should be looked at as the outcome of specific interactions between pathogen and host cells. Indeed, factors on both pathogen and host sides may be involved in the progression of the infection. In this context, it is widely accepted that host innate immunity plays a key role to clear *K. pneumoniae *infections. Therefore, differences among strains in the resistance to complement and/or to antimicrobial peptides mediated killing may account for differences in virulence [11,15,39]. In addition, a wealth of evidence clearly indicates the importance of the inflammatory responses in clearing *K. pneumoniae *infection and have provided substantial evidence for the protective role of a Th1-mediated response [40-42]. Thus, differences in the induction of inflammatory responses among strains may also underline *in vivo *behavior. In summary the available data support the notion that CPS-dependent cytotoxicity, together with other bacterially triggered events, is required for virulence. Further studies will attempt to elucidate these novel virulence mechanisms, which may differ among capsulated strains, in order to achieve a comprehensive understanding of *K. pneumoniae *pathogenesis.

## Conclusion

This study allocates a novel role to *K. pneumoniae *capsule, i.e. the induction of cytotoxicity during the infection of lung epithelial cells. This effect, which has been analysed by using four different approaches, is not capsule serotype dependent, does require the presence of live bacteria, and does not seem to be directly related to bacterial adhesion. Host cell cytotoxicity could be associated with virulence. However, strains expressing different capsule levels were not equally virulent, suggesting that additional bacterial elements could be involved in *Klebsiella *virulence.

## Authors' contributions

VC carried out the experiments involving lung epithelial cells infections. DM and ELL carried out the animal experiments. JAB. and JG conceived the study and wrote the manuscript. All authors read and approved the final version of the manuscript.

## References

[B1] Carpenter JL *Klebsiella *pulmonary infections: occurrence at one medical center and review Rev Infect Dis 1990 12 672 682 2201068 10.1093/clinids/12.4.672

[B2] Gupta A Hospital-acquired infections in the neonatal intensive care unit-*Klebsiella pneumoniae* Semin Perinatol 2002 26 340 345 10.1053/sper.2002.36267 12452506

[B3] Jarvis WR Munn VP Highsmith AK Culver DH Hughes JM The epidemiology of nosocomial infections caused by *Klebsiella pneumoniae* Infect Control 1985 6 68 74 3882593 10.1017/s0195941700062639

[B4] Bartlett JG O'Keefe P Tally FP Louie TJ Gorbach SL Bacteriology of hospital-acquired pneumonia Arch Intern Med 1986 146 868 871 10.1001/archinte.146.5.868 3516102

[B5] Straus DC Production of an extracellular toxic complex by various strains of *Klebsiella pneumoniae* Infect Immun 1987 55 44 48 3539806 10.1128/iai.55.1.44-48.1987 PMC260278

[B6] Strauss E A symphony of bacterial voices [news] Science 1999 284 1302 1304 10.1126/science.284.5418.1302 10383312

[B7] Álvarez D Merino S Tomás JM Benedí VJ Albertí S Capsular polysaccharide is a major complement resistance factor in lipopolysaccharide O side chain-deficient *Klebsiella pneumoniae *clinical isolates Infect Immun 2000 68 953 955 10.1128/IAI.68.2.953-955.2000 10639470 PMC97229

[B8] Sahly H Podschun R Oelschlaeger TA Greiwe M Parolis H Hasty D Kekow J Ullmann U Ofek I Sela S Capsule impedes adhesion to and invasion of epithelial cells by *Klebsiella pneumoniae* Infect Immun 2000 68 6744 6749 10.1128/IAI.68.12.6744-6749.2000 11083790 PMC97775

[B9] Schembri MA Dalsgaard D Klemm P Capsule shields the function of short bacterial adhesins J Bacteriol 2004 186 1249 1257 10.1128/JB.186.5.1249-1257.2004 14973035 PMC344426

[B10] Schembri MA Blom J Krogfelt KA Klemm P Capsule and fimbria interaction in *Klebsiella pneumoniae* Infect Immun 2005 73 4626 4633 10.1128/IAI.73.8.4626-4633.2005 16040975 PMC1201234

[B11] Campos MA Vargas MA Regueiro V Llompart CM Albertí S Bengoechea JA Capsule polysaccharide mediates bacterial resistance to antimicrobial peptides Infect Immun 2004 72 7107 7114 10.1128/IAI.72.12.7107-7114.2004 15557634 PMC529140

[B12] Llobet E Tomás JM Bengoechea JA Capsule polysaccharide is a bacterial decoy for antimicrobial peptides Microbiology 2008 154 3877 3886 10.1099/mic.0.2008/022301-0 19047754

[B13] Regueiro V Campos MA Pons J Albertí S Bengoechea JA The uptake of a *Klebsiella pneumoniae *capsule polysaccharide mutant triggers an inflammatory response by human airway epithelial cells Microbiology 2006 152 555 566 10.1099/mic.0.28285-0 16436443

[B14] Regueiro V Moranta D Campos MA Margareto J Garmendia J Bengoechea JA *Klebsiella pneumoniae *increases the levels of Toll-like receptors 2 and 4 in human airway epithelial cells Infect Immun 2009 77 714 724 10.1128/IAI.00852-08 19015258 PMC2632040

[B15] Cortés G Álvarez D Saus C Albertí S Role of lung epithelial cells in defense against *Klebsiella pneumoniae *pneumonia Infect Immun 2002 70 1075 1080 10.1128/IAI.70.3.1075-1080.2002 11854185 PMC127765

[B16] Cortés G Borrell N de Astorza B Gómez C Sauleda J Albertí S Molecular analysis of the contribution of the capsular polysaccharide and the lipopolysaccharide O side chain to the virulence of *Klebsiella pneumoniae *in a murine model of pneumonia Infect Immun 2002 70 2583 2590 10.1128/IAI.70.5.2583-2590.2002 11953399 PMC127904

[B17] Westphal O Jann K Bacterial lipopolysaccharides extraction with phenol-water and further applications of the procedure Meth Carbohydrate Chem 1963 5 83 91

[B18] Hirschfeld M Ma Y Weis JH Vogel SN Weis JJ Cutting edge: repurification of lipopolysaccharide eliminates signaling through both human and murine toll-like receptor 2 J Immunol 2000 165 618 622 10878331 10.4049/jimmunol.165.2.618

[B19] Manthey CL Perera PY Henricson BE Hamilton TA Qureshi N Vogel SN Endotoxin-induced early gene expression in C3H/HeJ (Lpsd) macrophages J Immunol 1994 153 2653 2663 7521367

[B20] Bitter T Muir HM A modified uronic acid carbazole reaction Anal Biochem 1962 4 330 334 10.1016/0003-2697(62)90095-7 13971270

[B21] Rahn A Whitfield C Transcriptional organization and regulation of the *Escherichia coli *K30 group 1 capsule biosynthesis (*cps*) gene cluster Mol Microbiol 2003 47 1045 1060 10.1046/j.1365-2958.2003.03354.x 12581358

[B22] Díaz-Aparicio E Aragón V Marín C Alonso B Font M Moreno E Pérez-Ortiz S Blasco JM Díaz R Moriyón I Comparative analysis of *Brucella *serotype A and M and *Yersinia enterocolitica *O:9 polysaccharides for serological diagnosis of brucellosis in cattle, sheep, and goats J Clin Microbiol 1993 31 3136 3141 8308104 10.1128/jcm.31.12.3136-3141.1993 PMC266364

[B23] Boyd AP Grosdent N Totemeyer S Geuijen C Bleves S Iriarte M Lambermont I Octave JN Cornelis GR *Yersinia enterocolitica *can deliver Yop proteins into a wide range of cell types: development of a delivery system for heterologous proteins Eur J Cell Biol 2000 79 659 671 10.1078/0171-9335-00098 11089914

[B24] Straus DC Atkisson DL Garner CW Importance of a lipopolysaccharide-containing extracellular toxic complex in infections produced by *Klebsiella pneumoniae* Infect Immun 1985 50 787 795 3905614 10.1128/iai.50.3.787-795.1985 PMC261149

[B25] Shifrin Y Kirschner J Geiger B Rosenshine I Enteropathogenic *Escherichia coli *induces modification of the focal adhesions of infected host cells Cell Microbiol 2002 4 235 243 10.1046/j.1462-5822.2002.00188.x 11952640

[B26] Taieb F Nougayrede JP Watrin C Samba-Louaka A Oswald E *Escherichia coli *cyclomodulin Cif induces G2 arrest of the host cell cycle without activation of the DNA-damage checkpoint-signalling pathway Cell Microbiol 2006 8 1910 1921 10.1111/j.1462-5822.2006.00757.x 16848790

[B27] Kuwae A Ohishi M Watanabe M Nagai M Abe A BopB is a type III secreted protein in *Bordetella bronchiseptica *and is required for cytotoxicity against cultured mammalian cells Cell Microbiol 2003 5 973 983 10.1046/j.1462-5822.2003.00341.x 14641181

[B28] Shafikhani SH Morales C Engel J The *Pseudomonas aeruginosa *type III secreted toxin ExoT is necessary and sufficient to induce apoptosis in epithelial cells Cell Microbiol 2008 10 994 1007 10.1111/j.1462-5822.2007.01102.x 18053004 PMC10952005

[B29] Stepinska M Trafny EA Diverse type III secretion phenotypes among *Pseudomonas aeruginosa *strains upon infection of murine macrophage-like and endothelial cell lines Microb Pathog 2008 44 448 458 10.1016/j.micpath.2007.11.008 18221854

[B30] Bomberger JM Maceachran DP Coutermarsh BA Ye S O'Toole GA Stanton BA Long-distance delivery of bacterial virulence factors by *Pseudomonas aeruginosa *outer membrane vesicles PLoS Pathog 2009 5 e1000382 10.1371/journal.ppat.1000382 19360133 PMC2661024

[B31] Kesty NC Mason KM Reedy M Miller SE Kuehn MJ Enterotoxigenic *Escherichia coli *vesicles target toxin delivery into mammalian cells EMBO J 2004 23 4538 4549 10.1038/sj.emboj.7600471 15549136 PMC533055

[B32] Allen PM Roberts I Boulnois GJ Saunders JR Hart CA Contribution of capsular polysaccharide and surface properties to virulence of *Escherichia coli *K1 Infect Immun 1987 55 2662 2668 3312006 10.1128/iai.55.11.2662-2668.1987 PMC259958

[B33] St Geme J III Falkow S Capsule loss by *Haemophilus influenzae *type b results in enhanced adherence to and entry into human cells J Infect Dis 1992 165 S117 S118 1350299 10.1093/infdis/165-supplement_1-s117

[B34] Talbot UM Paton AW Paton JC Uptake of *Streptococcus pneumoniae *by respiratory epithelial cells Infect Immun 1996 64 3772 3777 8751928 10.1128/iai.64.9.3772-3777.1996 PMC174292

[B35] Lawlor MS Hsu J Rick PD Miller VL Identification of *Klebsiella pneumoniae *virulence determinants using an intranasal infection model Mol Microbiol 2005 58 1054 1073 10.1111/j.1365-2958.2005.04918.x 16262790

[B36] Struve C Forestier C Krogfelt KA Application of a novel multi-screening signature-tagged mutagenesis assay for identification of *Klebsiella pneumoniae *genes essential in colonization and infection Microbiology 2003 149 167 176 10.1099/mic.0.25833-0 12576590

[B37] Struve C Krogfelt KA Role of capsule in *Klebsiella pneumoniae *virulence: lack of correlation between *in vitro *and *in vivo *studies FEMS Microbiol Lett 2003 218 149 154 10.1111/j.1574-6968.2003.tb11511.x 12583911

[B38] Sahly H Keisari Y Crouch E Sharon N Ofek I Recognition of bacterial surface polysaccharides by lectins of the innate immune system and its contribution to defense against infection: the case of pulmonary pathogens Infect Immun 2008 76 1322 1332 10.1128/IAI.00910-07 18086817 PMC2292847

[B39] de Astorza B Cortés G Crespí C Saus C Rojo JM Albertí S C3 promotes clearance of *Klebsiella pneumoniae *by A549 epithelial cells Infect Immun 2004 72 1767 1774 10.1128/IAI.72.3.1767-1774.2004 14977986 PMC356012

[B40] Greenberger MJ Kunkel SL Strieter RM Lukacs NW Bramson J Gauldie J Graham FL Hitt M Danforth JM Standiford TJ IL-12 gene therapy protects mice in lethal *Klebsiella pneumonia* J Immunol 1996 157 3006 3012 8816409

[B41] Standiford TJ Wilkowski JM Sisson TH Hattori N Mehrad B Bucknell KA Moore TA Intrapulmonary tumor necrosis factor gene therapy increases bacterial clearance and survival in murine gram-negative pneumonia Hum Gene Ther 1999 10 899 909 10.1089/10430349950018300 10223724

[B42] Ye P Garvey PB Zhang P Nelson S Bagby G Summer WR Schwarzenberger P Shellito JE Kolls JK Interleukin-17 and lung host defense against *Klebsiella pneumoniae *infection Am J Respir Cell Mol Biol 2001 25 335 340 11588011 10.1165/ajrcmb.25.3.4424

